# A rare case of *Mycobacterium Chelonae* infection in an immunocompromised adult with cavernous sinus syndrome

**DOI:** 10.1111/cns.13808

**Published:** 2022-02-15

**Authors:** Ye Li, Le Zhang, Chunhui Li, Xuelun Zou, Yi Zeng, Zhiping Hu, Bo Xiao, Lili Long

**Affiliations:** ^1^ Department of Neurology Xiangya Hospital Central South University Changsha China; ^2^ National Clinical Research Center for Geriatric Disorders Xiangya Hospital Central South University Changsha China; ^3^ Infection Control Center Xiangya Hospital Central South University Changsha China; ^4^ Department of Geriatrics Second Xiangya Hospital Central South University Changsha China; ^5^ Department of Neurology Second Xiangya Hospital Central South University Changsha China

**Keywords:** cavernous sinus syndrome, *Mycobacterium chelonae*, next‐generation sequencing, non‐tuberculous mycobacteria, case report

## CONFLICT OF INTEREST

The authors declare that the research was conducted in the absence of any commercial or financial relationships that could be construed as a potential conflict of interest.

## AUTHOR CONTRIBUTIONS

Y.L. and L.L. wrote the manuscript. Y.L., L.L., and C.L. were the physicians who treated the patient and provided professional comments to the manuscript. Y.L., L.Z., Z.H., Y.Z., X.Z., and B.X. analyzed the clinical data and the NGS data. All authors have read and approved the final manuscript.

Dear Editor,

*Mycobacterium chelonae* is a non‐tuberculous mycobacterium (NTM) that primarily causes skin and soft tissue infections in immunocompromised adults.^1^ Herein, we report a rare case of cavernous sinus syndrome (CSS) caused by *M*.* chelonae* infection in an immunocompromised adult female, which led to focal stenosis of the adjacent internal carotid artery (ICA) and pseudoaneurysm formation.

**FIGURE 1:**
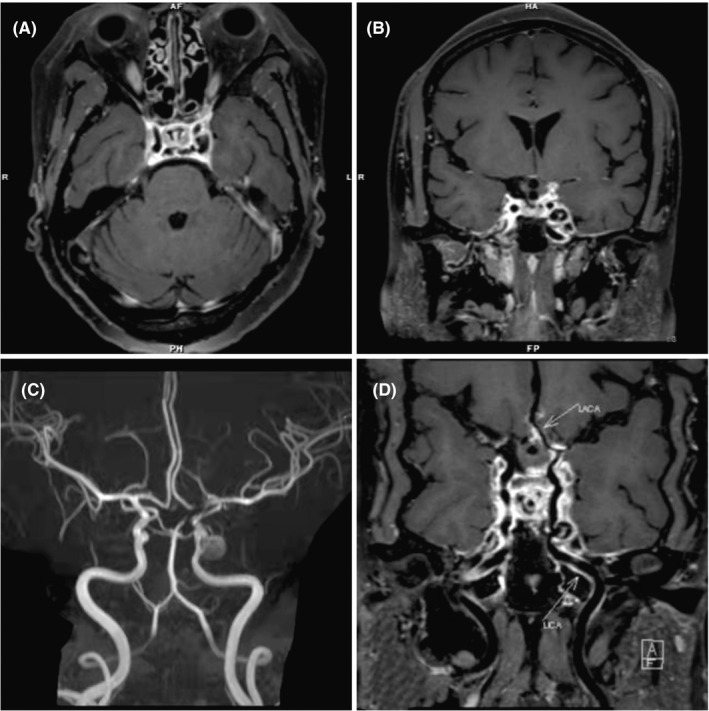
Brain imaging presentations. Axial and coronal T1 contrast‐enhanced MRI scan showing an enlargement of the left cavernous sinus (CS) with an enhancing lesion, an infectious pseudoaneurysm of the Lacerum segment of the left ICA, enhancing lesions encase the left internal carotid artery (ICA), which is narrowed (panel A and B). Three‐dimensional time of flight magnetic resonance angiograms (3D‐TOF MRA) suggests a focal narrowing of the adjacent ICA, the left ICA, left anterior cerebral artery, and left posterior cerebral artery, and Lacerum segment of the left ICA infectious pseudoaneurysm formation (panel C). High‐resolution vessel wall imaging after intravenous contrast injection revealed concentric wall thickening and enhancement of the left ICA, left anterior cerebral artery, and left posterior cerebral artery, compatible with inflammatory vasculopathy (panel D)

On July 4, 2020, a 43‐year‐old woman was admitted to a local hospital with fever and headache. Her symptoms began with peristomal herpes 3 days earlier. Lumbar puncture (LP) showed high levels of leukocytes (54 cells/µL, polymorphonuclear cells were slightly dominant) and a protein concentration of 663 mg/dl in cerebrospinal fluid (CSF) samples. Magnetic resonance imaging (MRI) and MR angiography of the brain revealed no abnormalities. Her symptoms eased within 2 weeks of antiviral and empirical antibiotic therapy. However, starting July 20, she had recurrent febrile episodes. Considering continued suspicion of immune response secondary to central nervous system (CNS) infection, dexamethasone (40 mg/day), a hormone immunoregulatory drug, was added to the treatment regime. On the sixth day after dexamethasone administration, the patient developed sixth nerve palsy (abduction deficit in the left eye) and loss of pinprick and thermal sensations in the territories of the ophthalmic (V1) and maxillary (V2) divisions of the left trigeminal nerve. MRI revealed enlargement of the left cavernous sinus (CS) with an enhancing lesion, secondary infectious arteritis, and infectious pseudoaneurysm formation (Figure 1). Thus, a clinical diagnosis of CSS was made. Multiple tests for potential autoimmune and neoplastic causes came back negative. After her fever subsided, the patient was discharged on August 5. However, she still had clinical manifestations of CSS.

On September 6, she was transferred to our hospital. She underwent LP for CSF sample analysis, which demonstrated a leukocyte count of 70 cells/mm^3^ (58% lymphocytes), a protein level of 380 mg/dl, and a glucose level of 2.78 mg/dl. Microbial culture results of the CSF samples were negative for bacteria, including mycobacteria, and fungi. However, the CSF sample was positive for modified acid‐fast staining. Moreover, negative results of the purified protein derivative and tuberculosis infection T‐cell spot test (T‐SPOT. TB) and lung CT may not necessarily suggest tuberculosis infection. She had no other laboratory abnormalities.

**FIGURE 2:**
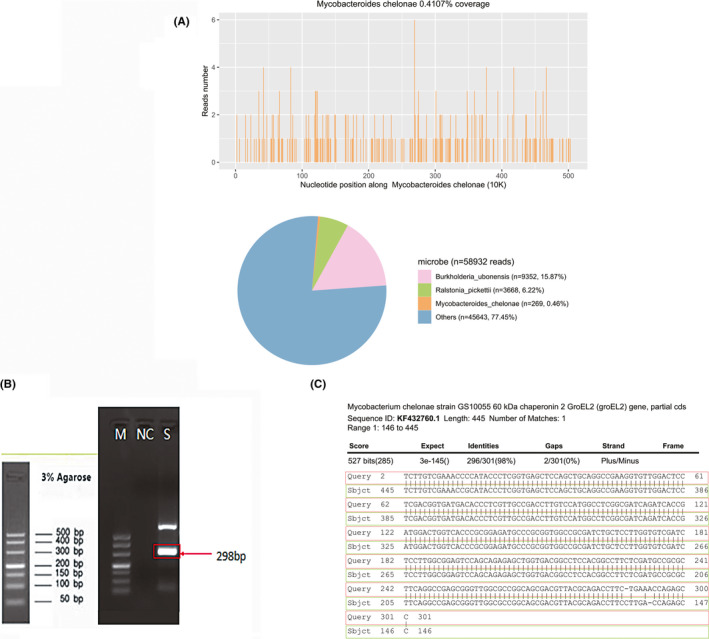
Diagnosis of *Mycobacterium chelonae* infection using the NGS and following verification using PCR and Sanger sequencing. Panel A: Mapping of *M*.* chelonae* reads to the genome and microbial community in CSF sample. PCR targeting heat‐shock protein 65 (*hsp65*) gene with specific primers (forward primer: GGCTCTGGTCAAGGAAGGTC, reverse primer: CTTGTCGAACCGCATACCCT) detected 298 base‐pair products in CSF sample and nothing in the control (panel B). Panel C: NCBI sequence alignment result. The red boxes indicate the Sanger sequencing result of the PCR product, and the green boxes show the reference sequence from NCBI database. The identity between the sequence obtained from Sanger sequencing and a reference *M*.* chelonae* sequence (GenBank accession no. KF432760.1) was 98%

CSF samples were collected for next‐generation sequencing (NGS) analysis on Day 4 of hospitalization. Libraries were multiplexed and sequenced using an Illumina platform, along with water and CSF from an uninfected clinical sample as negative controls (Appendix S1). Written consent for NGS analysis of clinical samples was obtained from the patient. *M*.* chelonae* was detected in the CSF from the initial rapid sequencing run completed on Day 6 of hospitalization. Sequencing identified 269 (out of 15,214,873) sequence reads uniquely corresponding to the *M*.* chelonae* genome (Figure 2A), and these reads covered a high percentage of the genome. When the reads from the human host were excluded, *M*.* chelonae* reads accounted for 0.46% of total microbial and unknown or unclassified reads (Figure 2A). NGS analysis performed on the CSF sample collected on Day 21 of hospitalization identified 40 (out of 17,569,288) sequence reads uniquely corresponding to the *M*.* chelonae* genome. The detection of *M*.* chelonae* in this subject was also confirmed by PCR and Sanger sequencing (Figure 2B,C; Figure S1).

The aforementioned results indicated that the patient was virtually infected with *M*.* chelonae*. She was then started on intravenous linezolid and cefoxitin, followed by oral doxycycline. Her symptoms gradually improved, and an MRI scan at 3 weeks showed that the enhancing lesion in the left CS had significantly reduced in size. LP suggested that the intracranial infection was largely improved, and a repeat NGS analysis of the CSF sample indicated that the copy number of *M*.* chelonae* had indeed decreased (40 reads). The patient was discharged 3 weeks later, with a dramatic response to therapy.

At the 1‐month follow‐up, contrast‐enhanced scans showed no abnormal enhancement in the CS areas. At the 3‐month follow‐up, all CSF parameters had returned to normal. Examination at 6 months showed no apparent positive signs.

In this study, we used NGS analysis to identify a rare case of CSS caused by *M*.* chelonae* infection. NTM infections are always challenging and involve the lungs, skin, soft tissue, and CNS.^2^ Although NTM CNS infections are rare, the mortality rate is high.^3^ Infections due to this pathogen are on the rise, and skin and soft tissue infections have been reported, involving skeletal tissue, eye, ear, lung, and rare cases of endocarditis and meningitis.^1^ *Mycobacterium chelonae* is an opportunistic pathogen that infects immunocompromised individuals.^4^ Therefore, in the present case, the patient's previous history of glucocorticoid therapy cannot be ignored. Empirical treatment with corticosteroids often clouds the diagnosis of CNS infection, as it transiently improves systemic features and occasionally improves focal neurological signs, but later accelerates the progression of infection.^5^

Cavernous sinus is a complex venous space surrounded by the dural folds and contains important neurovascular structures (III, IV, and VI nerves; first and second trigeminal branches; ICA, sympathetic plexus). The valveless venous communication system comprises almost every important venous structure in the head and neck. Different pathologic conditions, such as infectious, inflammatory, vascular, and neoplastic diseases, can give rise to CSS with unique clinical presentations. Although infectious and inflammatory etiologies are difficult to distinguish using imaging data, clinical symptoms and laboratory tests can help narrow down the differential diagnosis, which includes Tolosa‐Hunt syndrome, sarcoidosis, granulomatosis with polyangiitis, IgG‐4‐related disease, and invasive fungal infections, such as aspergillosis and mucormycosis.^6^ However, to the best of our knowledge, CSS caused by *M*.* chelonae* infection has not been reported previously. In suspected cases, close attention should be paid to assess secondary infectious vasculitis and infectious pseudoaneurysm formation.

In the present case, NGS analysis played a critical role in the accurate diagnosis of *M*.* chelonae* infection. Hence, NGS can be a valuable tool in the diagnosis of CNS infection, as it can detect causative pathogens even when routine tests fail to do so.^7^ However, the core challenge with regard to using NGS is to develop faster, more accurate, and more complete identification of pathogens in a large human background and ubiquitous bacteria background. In addition, for different pathogens detected by NGS, it is worth further discussion to determine the diagnostic value of their sequence number. In the present case, we could only determine *M*.* chelonae* infection after comprehensive consideration of all analyses, including the results of the patient's two NGS analyses, Sanger sequencing, combined with the patient's clinical manifestations, imaging, CSF characteristics, responses to treatment, and outcome. In the future, the NGS sequence number of *M*.* chelonae* and other pathogens in the CSF may indicate that CNS infection needs to be studied in a larger sample size.

## Supporting information

Fig S1

Appendix S1

## Data Availability

The datasets presented in this study can be found in online repositories. The names of the repository and accession number can be found below: National Genomics Data Center, accession No: PRJCA007940.
