# Supplementary figures and images for: A rare case of Mycobacterium Chelonae infection in an immunocompromised adult with cavernous sinus syndrome
Source: CNS Neurosci Ther. 2022 Feb 15;28(5):796–9. doi: 10.1111/cns.13808 (PMC8981426; doi:10.1111/cns.13808)

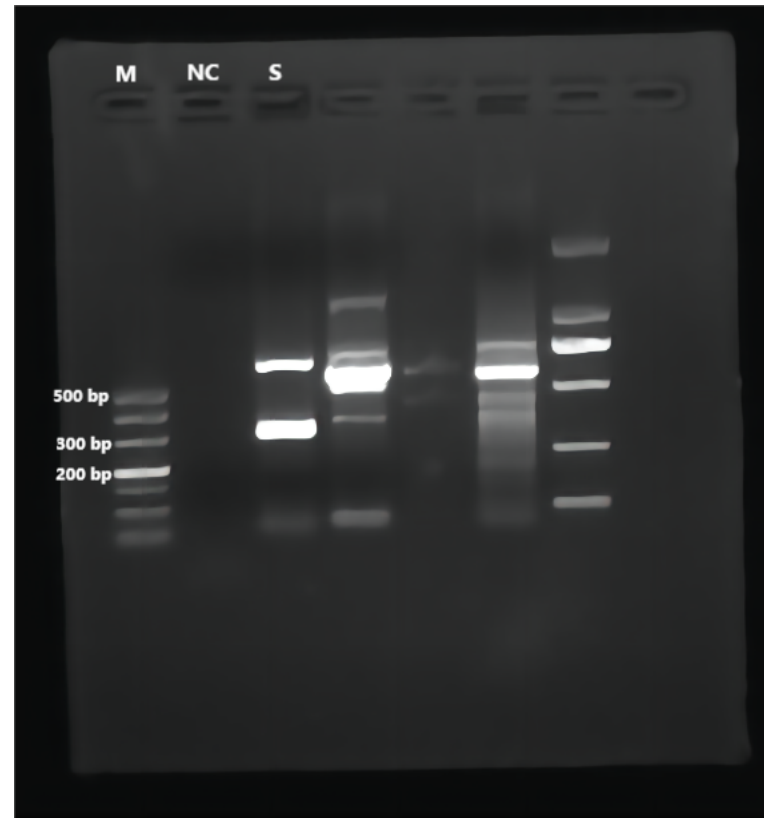

Figure. Full unedited gel for figure 2B

Supplement: Supplementary file 1 — Fig S1 [file CNS-28-796-s002.pdf]
