# Supplementary material for: A rare case of Mycobacterium Chelonae infection in an immunocompromised adult with cavernous sinus syndrome
Source: CNS Neurosci Ther. 2022 Feb 15;28(5):796–9. doi: 10.1111/cns.13808 (PMC8981426; doi:10.1111/cns.13808)
Supplement: Supplementary file 2 — Appendix S1 [file CNS-28-796-s001.doc]

**Supplementary Appendix**

**Metagenomic Next-generation Sequencing and Analysis**

**Methods**

**Sample Collection and Nucleic Acid Extraction**

About 2-3 mL cerebrospinal fluid (CSF) was collected and sealed using sterile technique after patient enrolled, and then stored below -20℃ or shipped on dry ice to Hugobiotech Co., Ltd., (Beijing, China) to perform mNGS detection immediately. The DNA was extracted and purified from 200 uL CSF supernatant according to the manufacture’s instruction of TIANGEN DNA Mini kit DP316. DNA concentration and quality were checked through Qubit and agarose gel electrophoresis.

**Library Generation and Sequencing**

The DNA libraries was constructed using QIAseq™ Ultralow Input Library Kit. The concentration and quality of libraries were checked using Qubit and agarose gel electrophoresis. Qualified libraries with different barcode labeling were pooled together, and then sequenced on an Illumina Nextseq platform.

**Bioinformation Pipeline**

After obtaining the sequencing data, High-quality data were generated after filtering out adapter, low-quality, low-complexity, and shorter reads. Next, remove human reads by mapping reads to human reference genome using SNAP software. The remaining data were aligned to the microbial genome database using Burrows-Wheeler Alignment. The database collected microbial genomes from NCBI. It contains more than 20,000 microorganisms, including 11910 bacteria, 7103 viruses, 1046 fungi and 305 parasites. Finally get the microbial compositions of the sample.
